# A functional genomics approach to identify pathways of drug resistance in medulloblastoma

**DOI:** 10.1186/s40478-018-0652-8

**Published:** 2018-12-27

**Authors:** Kelsey C. Bertrand, Claudia C. Faria, Patryk Skowron, Amanda Luck, Livia Garzia, Xiaochong Wu, Sameer Agnihotri, Christian A. Smith, Michael D. Taylor, Stephen C. Mack, James T. Rutka

**Affiliations:** 1 Department of Pediatrics, Division of Hematology and Oncology, Baylor College of Medicine, Houston, USA; 2 Brain Tumor Program, Cancer and Hematology Centers, Texas Children’s Hospital, Houston, USA; 3 Department of Neurosurgery, Hospital de Santa Maria, Centro Hospitalar Lisboa Norte, EPE, Lisbon, Portugal; 4 Department of Developmental and Stem Cell Biology and Regenerative Medicine, The Hospital for Sick Children, Toronto, Canada; 5 Arthur and Sonia Labatt Brain Tumor Research Center, The Hospital for Sick Children, Toronto, Canada; 6 Department of Laboratory Medicine and Pathobiology, University of Toronto, Toronto, Canada; 7 Department of Neurosurgery, The Hospital for Sick Children, Toronto, Canada; 8 Department of Neurological Surgery, Children’s Hospital, University of Pittsburgh School of Medicine, Pittsburgh, USA

**Keywords:** Cancer, Brain tumor, Medulloblastoma, Drug resistance, Functional genomics, Transposon mutagenesis, Sleeping beauty

Medulloblastoma (MB) is the most common malignant pediatric brain tumor [10]. Although continued advances have been made in our understanding of medulloblastoma, questions remain about its etiology and treatment [3, 4, 7, 9, 13–15, 17, 18]. Medulloblastoma is classified into four demographically, clinically, and molecularly distinct subgroups called WNT, SHH, Group 3 and Group 4 (As reviewed in [19]), and further divided into additional distinct molecular subtypes [3, 14]. While patients with (WNT) pathway driven medulloblastoma have favorable outcomes, recurrence rates are higher in other subtypes such as SHH, which tend to recur locally, and Group 3 and 4, which are associated with distal metastases [16]. The biology of metastatic medulloblastoma is distinct from primary medulloblastoma; representing potentially a different therapeutic disease [6, 8, 13]. Approaches are needed to identify pathways of therapy resistance in both primary and metastatic compartments of medulloblastoma to guide selection of targeted therapies. As an example, the MET proto-oncogene is upregulated in SHH and Group 3 medulloblastoma, and can be targeted by treatment of cells and mouse models with the small molecule inhibitor, Foretinib [4]. We utilized a spontaneous metastatic mouse model of medulloblastoma driven by the Sleeping Beauty mutagenesis transposon system [21] to pinpoint functional drivers and pathways of resistance to Foretinib [4] in both primary and metastatic medulloblastoma. This serves as a novel approach to dissecting patterns of therapy resistance at multiple tumor sites simultaneously which can be readily applied to other cancer systems.

## Approach

**Fig. 1:**
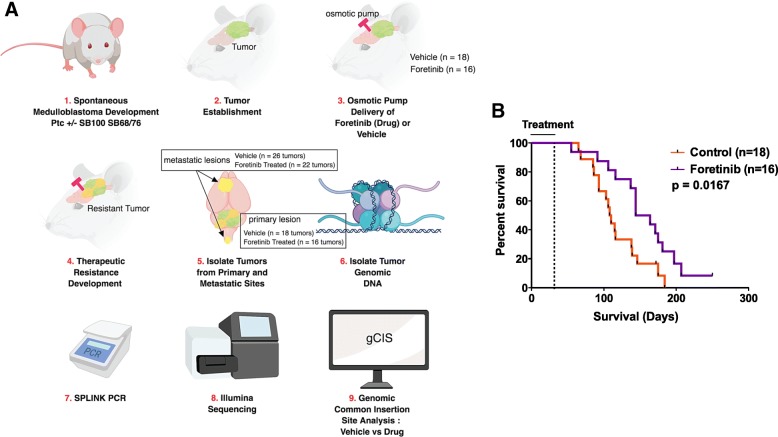
A transposon-mutagenesis system to identify genes mediating Foretinib resistance in Medulloblastoma. **a** A schematic describing the steps used to treat medulloblastoma-bearing mice with Foretinib and the identification of transposon insertion sites. **b** A Kaplan-Meier plot demonstrating significant improvement in overall survival in mice receiving Foretinib treatment

A medulloblastoma sleeping beauty transposon mutagenesis mouse model (*Ptch*+/−, SB100/SB68, T2Onc), that spontaneously develops primary and metastatic medulloblastoma with 100% penetrance, was used to identify pathways of Foretinib resistance [13] (Fig. 1a). This system allows for entrapment of both oncogenes and tumor suppressor genes, which can be identified by next-generation sequencing of transposon insertion sites [21]. We showed previously that Foretinib is an effective inhibitor of the MET pathway in SHH and Group 3 medulloblastoma mouse models [4] (Fig. 1b). Following tumor establishment in a time frame of 30–35 days, mice were treated with vehicle or Foretinib, through continuous osmotic pump infusion into the cerebrospinal fluid for 28 days at a rate of 0.25 μl per hour (Fig. 1a). Resistant primary and metastatic tumors were harvested and genomic common insertion sites (gCIS) were identified by SPLINK PCR combined with paired-end Illumina high-throughput sequencing (Fig. 1a), to pinpoint genetic drivers of therapy resistance.

## Results and discussion

**Fig. 2:**
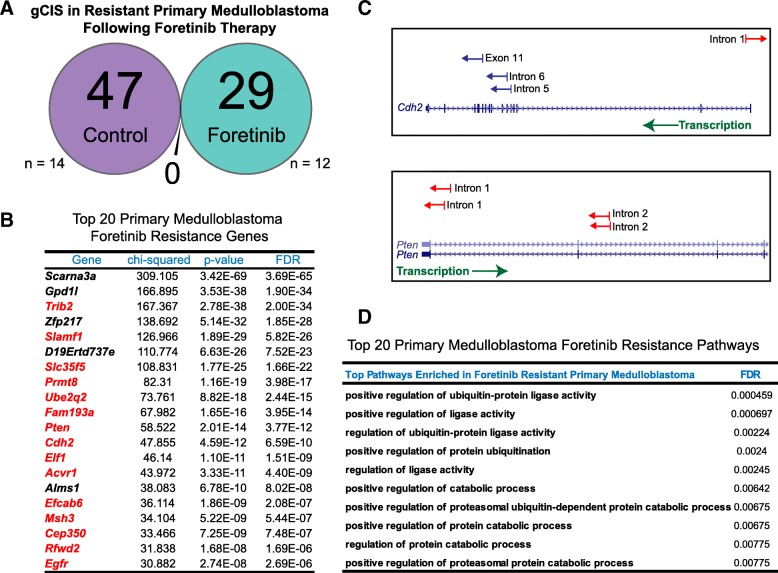
Transposon insertion patterns are divergent in primary medulloblastoma receiving Foretinib therapy. **a** A Venn diagram illustrating the number of statistically significant gCISs exclusive or shared gCISs between vehicle (*n* = 14) and Foretinib (*n* = 12) treated primary medulloblastoma. **b** A table showing the Top 20 statistically significant Foretinib resistance genes in primary medulloblastoma. Highlighted in red are genes which have been reported to be mutated in cancer when compared against the COSMIC database. **c** Examples of transposon insertions in *Cdh2* and *Pten* and their direction of orientation (red = anti-sense, blue = sense) relative to direction transcription (green). **d** Pathway analysis of Foretinib-resistance genes in primary medulloblastoma identified using GeneMania

We leveraged the Sleeping Beauty transposon system to identify pathways of resistance to Foretinib as a proof-of-concept strategy, which could be applied to other cancer models and systems. In primary medulloblastoma, the patterns of gCISs in Foretinib-treated mice were highly distinct from control mice, thus supporting that the underlying mechanisms of primary medulloblastoma are different from tumors receiving Foretinib therapy (Fig. 2a). In primary Foretinib-treated tumors, we identified specific insertions in known tumor oncogenes and tumor suppressor genes such as *Pten*, *Cdh2*, *Egfr*, and *Acvr1b* [1, 5, 20] (Fig. 2a-c). As two examples, *PTEN* insertions were predicted to being inactivating mutations by their location and antisense orientation within the 5′ end of the gene introducing an early poly A transcriptional termination signal. *CDH2* insertions were predicted to either promote expression of small isoforms of CDH2 and/or ablate expression of long isoforms of the gene. The majority of these candidates have been shown to be mutated in cancer when compared against the Catalogue of Somatic Mutations in Cancer database and several have been previously implicated in brain tumorigenesis [1, 2, 5, 20] (Fig. 2b). Further, the mechanisms mediating Foretinib-resistance in primary site tumors were over-represented by pathways involved in protein metabolism, specifically ubiquitin-mediated protein degradation (Fig. 2d). Our findings demonstrate that mice bearing medulloblastoma and receiving Foretinib therapy exhibit distinct pathway alterations from primary lesions. These pathways, identified through Sleeping Beauty transposon insertion analysis, represent candidate drivers and potential targets in Foretinib resistant medulloblastoma.

**Fig. 3:**
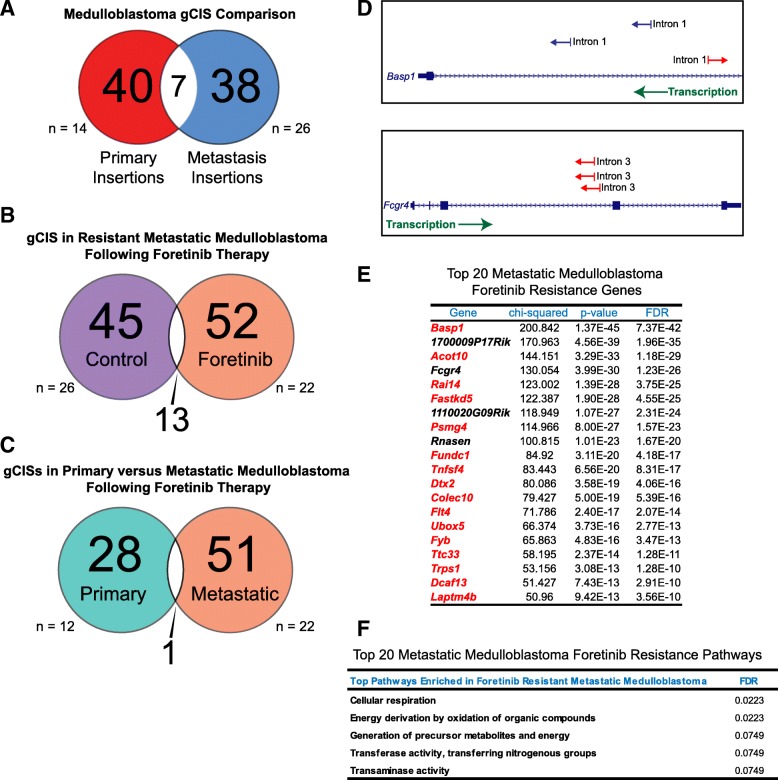
Divergent patterns of transposon insertions in metastatic medulloblastoma following Foretinib therapy. **a** A Venn diagram illustrating the number of statistically significant gCISs identified as exclusive or shared between primary (*n* = 14) and metastatic medulloblastoma (*n* = 26). **b** A Venn diagram illustrating the number of statistically significant gCISs exclusive or shared gCISs between vehicle (*n* = 26) and Foretinib treated metastatic medulloblastoma (*n* = 22). **c** A Venn diagram comparing the gCISs between primary (*n* = 12) and metastatic (*n* = 22) Foretinib treated medulloblastoma. **d** A table showing the Top 20 statistically significant Foretinib resistance genes in metastatic medulloblastoma. Highlighted in red are genes, which have been reported to be mutated in cancer when compared against the COSMIC database. **e** Examples of transposon insertions in *Basp1* and *Fcgr4* and their direction of orientation (red = anti-sense, blue = sense) relative to direction transcription (green). **f** Pathway analysis of Foretinib-resistance genes in metastatic medulloblastoma identified using GeneMania

Historically, metastatic disease has been assumed to be highly similar to primary tumors, and therefore presumably equally responsive to treatments designed to target primary lesions. Using the Sleeping Beauty Transposon system, we show that primary and metastatic medulloblastoma exhibit distinct patterns of genetic alterations (Fig. 3a, Additional file 1: Table S1-S4). gCISs identified in primary medulloblastoma included transcriptional regulators such as *Crebbp*, and *Ep300*, and in metastatic medulloblastoma immune response-related genes such as *C6*, *A2m*, and *Pkp2* (Additional file 1: Table S5-S8). These data support that the primary and metastatic compartments of medulloblastoma are driven by distinct molecular mechanisms [12]. We next asked whether metastatic medulloblastoma might evolve different or convergent pathways of resistance, as compared to the primary-treated tumors. We found that metastatic medulloblastoma receiving Foretinib therapy exhibited distinct patterns of genomic insertions compared to the metastatic compartment of vehicle treated mice (Fig. 3b). Furthermore, metastatic gCISs were highly divergent from the primary compartment in mice, which had also received Foretinib therapy (Fig. 3c). Foretinib-resistant metastatic medulloblastoma insertions included *Basp1*, *Flt4*, *Mllt10*, and *Asxl2* (Fig. 3d,e) and pathways involved in cellular metabolism (Fig. 3f). These findings demonstrate that primary and metastatic medulloblastoma are molecularly distinct; hence their response and resistance to therapy may be highly divergent. Furthermore we demonstrate the effectiveness of functional genomic mapping to simultaneously identify putative drivers of tumorigenesis in distinct tumor compartments.

## Conclusion

Our study has identified potential pathways that medulloblastoma cells may co-opt to overcome Foretinib inhibition, and provides a strategy for which drug resistance pathways to other medulloblastoma targeted therapies may be identified. Prospective identification of these pathways could be used to determine combinatory treatments that may be effective for resistant primary and metastatic tumor clones. We further demonstrate in our model that primary and metastatic medulloblastoma are genetically distinct, and in response to Foretinib-therapy, exhibit divergent mechanisms of resistance. A limitation of our method is that while driver pathways may be identified, they may not represent the exact genes targeted in resistant human primary tumors. Therefore, integrative functional mouse modeling using this Sleeping Beauty Approach paired with genomic characterization of resistant primary tumors, may prioritize pathways and specific targets that mediate cancer therapy resistance. Finally, our data lends support that treatments armed against genetic targets in the primary site may be ineffective for metastatic lesions, and that potentially distinct genetic evolution occurs between primary and metastatic medulloblastoma under therapy.

## Materials and methods

### Animal studies

All mouse studies were approved and performed in accordance to the policies and regulations of the Institutional Animal Care and Use Committee of the University of Toronto and the Hospital for Sick Children. A medulloblastoma Sleeping Beauty transposon mutagenesis murine model (*Ptch*^*+/−*^*/SB11*/T2Onc) was used, which frequently and spontaneously develops primary and metastatic MB. *Ptch*^*+/−*^*/SB11*/T2Onc mice were generously provided by Dr. Michael D. Taylor, Hospital for Sick Children, Toronto, Canada. Mice at post-natal day 30–35 were treated with vehicle or Foretinib (6 mg/kg), via Alzet osmotic pump (Model 2004) slow-infusion into the cerebrospinal fluid of the right lateral ventricle, for 28 days at a rate of 0.25ul/hour. Nucleic acid extractions were carried out as previously described. Statistical differences in survival curves of mice was assessed using a Kaplan-Meier estimate and log-rank test.

### Splinkerette PCR and common genomic insertion site analysis

Transposon common insertion sites were identified by SPLINK PCR of tumour DNA, followed by 100 bp paired-end Illumina next-generation sequencing (HiSeq 2500). Genomic DNA was digested and ligated with linker +/− primers, amplified through PCR, then further amplified with barcoded primers through a second PCR. DNA was then purified and prepared for sequencing; protocol as previously described [10]. Gene pathways were identified by querying gene lists with GeneMania [11], and significance measured using a hypergeometric distribution test. Data availability: The datasets supporting the conclusions of this article are included within this article. Raw data will be made openly available through the GEO repository.

## Additional file

Additional file 1: **Table S1.** Control Insertions - Primary Medulloblastoma. **Table S2.** Control Insertions - Primary Medulloblastoma (per sample). **Table S3.** Foretinib Specific Insertions - Primary Medulloblastoma. **Table S4.** Foretinib Insertions - Primary Medulloblastoma (per sample). **Table S5.** Control Insertions - Metastatic Medulloblastoma. **Table S6.** Control Insertions - Metastatic Medulloblastoma (per sample). **Table S7.** Foretinib Specific Insertions - Metastatic Medulloblastoma. **Table S8.** Foretinib Insertions - Metastatic Medulloblastoma (per sample). (XLSX 479 kb)

## References

[1] Brennan CW (2013). The somatic genomic landscape of glioblastoma. Cell.

[2] Buczkowicz P (2014). Genomic analysis of diffuse intrinsic pontine gliomas identifies three molecular subgroups and recurrent activating ACVR1 mutations. Nat Gen.

[3] Cavalli FMG (2017). Intertumoral Heterogeneity within Medulloblastoma Subgroups. Cancer cell.

[4] Faria CC (2015). Foretinib is effective therapy for metastatic sonic hedgehog medulloblastoma. Cancer Res.

[5] Fontebasso AM et al (2014) Recurrent somatic mutations in ACVR1 in pediatric midline high-grade astrocytoma. 46:462–466. 10.1038/ng.2950 10.1038/ng.2950 PMC4282994 24705250

[6] Genovesi LA (2013). Sleeping Beauty mutagenesis in a mouse medulloblastoma model defines networks that discriminate between human molecular subgroups. Proc Natl Acad Sci U S A.

[7] Hovestadt V (2014). Decoding the regulatory landscape of medulloblastoma using DNA methylation sequencing. Nature.

[8] Jenkins NC (2014). Genetic drivers of metastatic dissemination in sonic hedgehog medulloblastoma. Acta Neuropathologica Commun.

[9] Kool M (2014). Genome sequencing of SHH medulloblastoma predicts genotype-related response to smoothened inhibition. Cancer cell.

[10] Louis DN (2007). The 2007 WHO classification of tumours of the central nervous system. Acta neuropathologica.

[11] Montojo J, Zuberi K, Rodriguez H, Bader GD, Morris Q (2014). GeneMANIA: Fast gene network construction and function prediction for Cytoscape. F1000Res.

[12] Morrissy AS (2016). Divergent clonal selection dominates medulloblastoma at recurrence. Nature.

[13] Northcott PA (2012). Subgroup-specific structural variation across 1,000 medulloblastoma genomes. Nature.

[14] Northcott PA (2017). The whole-genome landscape of medulloblastoma subtypes. Nature.

[15] Pugh TJ (2012). Medulloblastoma exome sequencing uncovers subtype-specific somatic mutations. Nature.

[16] Ramaswamy V (2013). Recurrence patterns across medulloblastoma subgroups: an integrated clinical and molecular analysis. Lancet Oncol.

[17] Robinson G (2012). Novel mutations target distinct subgroups of medulloblastoma. Nature.

[18] Shih DJ (2014). Cytogenetic prognostication within medulloblastoma subgroups. J Clin Oncol.

[19] Taylor MD (2012). Molecular subgroups of medulloblastoma: the current consensus. Acta Neuropathologica.

[20] Wu G (2014). The genomic landscape of diffuse intrinsic pontine glioma and pediatric non-brainstem high-grade glioma. Nat Gen.

[21] Wu X (2012). Clonal selection drives genetic divergence of metastatic medulloblastoma. Nature.

